# The Role of Thioredoxin System in *Shank3* Mouse Model of Autism

**DOI:** 10.1007/s12031-024-02270-y

**Published:** 2024-09-30

**Authors:** Wisam Bazbaz, Maryam Kartawy, Wajeha Hamoudi, Shashank Kumar Ojha, Igor Khaliulin, Haitham Amal

**Affiliations:** Institute of Drug Research, School of Pharmacy, Faculty of Medicine, The Hebrew University of Jerusalem, Jerusalem, Israel

**Keywords:** Autism spectrum disorder, Nitric oxide, Oxidative stress, Nitrosative stress, Developmental disorders, Thioredoxin, Peroxiredoxin, Neuroscience

## Abstract

Autism spectrum disorder (ASD) is a complex neurodevelopmental disorder characterized by difficulties in social interaction and communication, repetitive behaviors, and restricted interests. Unfortunately, the underlying molecular mechanism behind ASD remains unknown. It has been reported that oxidative and nitrosative stress are strongly linked to ASD. We have recently found that nitric oxide (NO•) and its products play an important role in this disorder. One of the key proteins associated with NO• is thioredoxin (Trx). We hypothesize that the Trx system is altered in the *Shank3* KO mouse model of autism, which may lead to a decreased activity of the nuclear factor erythroid 2-related factor 2 (Nrf2), resulting in oxidative stress, and thus, contributing to ASD-related phenotypes. To test this hypothesis, we conducted in vivo behavioral studies and used primary cortical neurons derived from the *Shank3* KO mice and human SH-SY5Y cells with *SHANK3* mutation. We showed significant changes in the levels and activity of Trx redox proteins in the *Shank3* KO mice. A Trx1 inhibitor PX-12 decreased Trx1 and Nrf2 expression in wild-type mice, causing abnormal alterations in the levels of synaptic proteins and neurotransmission markers, and an elevation of nitrosative stress. Trx inhibition resulted in an ASD-like behavioral phenotype, similar to that of *Shank3* KO mice. Taken together, our findings confirm the strong link between the Trx system and ASD pathology, including the increased oxidative/nitrosative stress, and synaptic and behavioral deficits. The results of this study may pave the way for identifying novel drug targets for ASD.

**Supplementary Information:**

The online version contains supplementary material available at 10.1007/s12031-024-02270-y.

## Introduction

Autism spectrum disorder (ASD) is a common neurodevelopmental and behavioral disorder (Happé et al. 2006), typically identified in early childhood. ASD is characterized by social and communication deficits, restricted and repetitive behaviors, and fixated interests (Xiaoyu Tong et al. 2024). The underlying causes of ASD are still unknown. Current prevalence estimates suggest that 1–3% of children are affected with this disorder (Christensen et al. 2019). Many clinical trials aimed at relieving the core and associated symptoms of ASD have been carried out or are still ongoing (Baribeau and Anagnostou 2022). However, despite all the efforts, ASD prevalence continues to grow, and no pharmacological treatment has been approved for the core ASD symptoms to date. The underlying causes of this disorder are still unknown.

Genetic factors play a key role in ASD. *SHANK3*, one of the top ASD high-risk genes (Bossolani-Martins et al. 2010), encodes the Shank3 protein, a scaffolding protein found in excitatory synapses (Wan et al. 2021). Shank3 regulates actin polymerization, dendritic spine morphology, and synaptic transmission (Durand et al. 2012). *SHANK3* mutations occur in only 1.1% of ASD cases. In addition, *Shank3* mutant mice exhibit autistic-like behaviors, including repetitive grooming and social deficits (Peça et al. 2011). Our previous studies have shown elevated nitric oxide (NO•) production, nitrosative stress, and S-nitrosylation (SNO), the reversible covalent bond formation between NO• and a cysteine thiol within a protein, in *Shank3* mouse models of ASD (Amal et al. 2020a; Kartawy et al. 2021). Moreover, we have found that inhibiting neuronal NO• synthase (nNOS) pharmacologically in *Shank3* KO mice can reverse the behavioral ASD-associated phenotype and the molecular and synaptic dysfunctions (Tripathi et al. 2023; Abdel-Haq et al. 2023). Additionally, our work highlights the crucial role of NO• in both physiological and pathological states within neurological diseases, particularly ASD (Amal et al. 2020a, 2020b, 2019; Kartawy et al. 2021, 2020; Tripathi et al. 2023, 2024, 2020, 2022; Abdel-Haq et al. 2023; Hamoudi et al. 2022, 2021; Kruglyakov et al. 2023; Yang et al. 2022; Mencer et al. 2021; Khaliulin et al. 2020; Steinert and Amal 2023).

Nitric oxide is a highly reactive free radical and neurotransmitter involved in key neurobiological processes like morphogenesis, synaptic plasticity, and behavior modulation (Kruglyakov et al. 2023; Bórquez et al. 2016; Džoljić et al. 2015). While NO•-mediated post-translational modifications (PTMs) regulate cell signaling, NO• overproduction leads to nitrosative and nitrative stress, forming compounds like S-nitrosoglutathione (GSNO) and peroxynitrite (ONOO–), along with PTMs like SNO and tyrosine nitration with the formation of 3-nitrotyrosine (3-Ntyr) (Bandookwala and Sengupta 2020), leading to neuronal damage (Zhan et al. 2015). In addition to nitrosative stress, increasing evidence suggests a role of oxidative stress in the development and clinical manifestation of ASD (Zoroglu et al. 2004; Chauhan and Chauhan 2006). Furthermore, several articles showed that autistic children have redox abnormalities (Goldani et al. 2014). The severity of oxidative/nitrosative stress depends on the of reactive oxygen species (ROS) and reactive nitrogen species (RNS) production on the one hand and the strength of the endogenous antioxidant system on the other hand. In pathological conditions, ONOO^−^, NO•, and oxygen free radicals can be generated at high levels that exceed the antioxidant detoxification capacity (Dawson and Dawson 1996). Consequently, the balance shifts towards oxidative stress. Oxidative stress may lead to altered synaptic function (Vajda 2002), neuronal death (Onyango et al. 2006), neuroinflammation (McDougle and Carlezon 2013), lipid peroxidation (Ming et al. 2005), protein and DNA oxidation, altered immune response, decreased DNA methylation, and epigenetic dysregulation, which collectivity may result in ASD (Fatemi et al. 2012; Bjørklund et al. 2020a).

The major antioxidant system in the brain is the thioredoxin (Trx) system consisting of Trx, Trx reductase (TrxR), and NADPH (Lu and Holmgren 2014). Studies have uncovered a complex crosstalk between NO and Trx, which regulates various redox-dependent pathways (Benhar 2015). Additionally, Trxs act as disulfide reductases (Rozell et al. 1985), reducing peroxiredoxins (Prdxs 1–6) to their active form, which detoxifies organic hydroperoxides and H₂O₂ (Benhar 2015; Lillig and Holmgren 2007; Zhang et al. 2017; Nordberg and Arnér 2001). Prdxs also serve as oxidative stress markers (Poynton and Hampton 2014). Trx and its redox enzymes play a key role in defending against nitrosative stress by reducing and denitrosylating many proteins (Thom et al. 2012; Benhar et al. 2008; Nikitovic and Holmgren 1996). Furthermore, Trx can also regulate NO• production by denitrosylating nNOS (Qu et al. 2012) and inducible NOS (iNOS) (Jakupoglu et al. 2005). The Trx/Prdx system also defends against oxidative/nitrosative stress by ROS scavenging (Graves et al. 2009).

Trx1, the most well-characterized thioredoxin protein, is found in the cytosol but can also be present in the nucleus (Lu and Holmgren 2014; Rozell et al. 1985). In the cytoplasm, Trx1 helps maintain redox homeostasis by reducing oxidative stress and neutralizing ROS (Wu et al. 2011). In the nucleus, it regulates transcription factors, gene expression, apoptosis, and cell survival (Hirota et al. 1997). Upon nuclear translocation, Trx1 activates the Nrf2 pathway, which responds to ROS by inducing the expression of over 250 genes involved in redox regulation, antioxidant defense, DNA repair, apoptosis, and autophagy (Dodson et al. 2019; Hasan et al. 2022).

Trx1 can be inhibited by PX-12, which reversibly oxidizes its active site (-Cys32-Gly-Pro-Cys35-) and irreversibly inactivates this enzyme by thioalkylating Cys73. PX-12 also inhibits TrxR1, blocking the disulfide reductase activity of the Trx system (Lundberg et al. 2019).

**Fig. 1:**
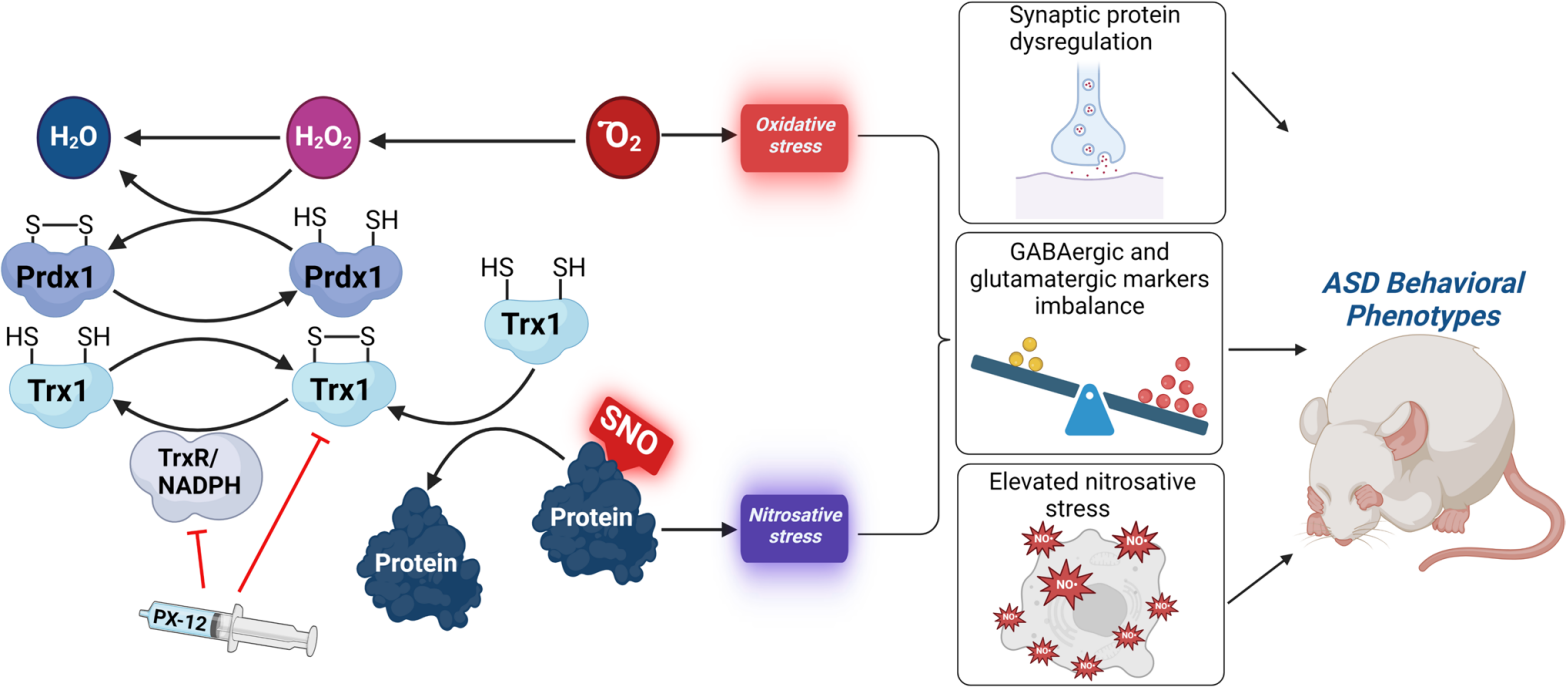
Schematic illustration of the role of the thioredoxin/peroxiredoxin system in ASD pathology. Pharmacological inhibition of Trx1 disrupts the balance of the thioredoxin/peroxiredoxin system leading to redox imbalance, synaptic protein alterations, neurotransmission marker imbalance, and induces ASD-like behavior

Accumulated evidence showed that Trx1 downregulation in the brain is observed in several neurological diseases, such as Alzheimer’s disease (Akterin et al. 2006), Parkinson’s disease (Arodin et al. 2014), and Huntington’s disease (Sánchez-López et al. 2012). However, the role of Trx1 in ASD pathology remains poorly understood. Therefore, in this study, we aimed to investigate the involvement of Trx1 in the *Shank3* KO mouse model of autism. We examined the effects of the Trx1 system inhibition on the activity of Nrf2, which affects the transcription of several redox proteins including Trx1 itself, and may lead to oxidative and nitrosative stress, and activity of its redox partner Prdx (Fig. 1). We also examined the contribution of the Trx system inhibition to the ASD-related phenotypes. To this end, we conducted several experiments using in vitro and in vivo models of *Shank3* mutations.

## Materials and Methods

### Materials

Primary antibodies, anti-thioredoxin 1 (#2298), anti-thioredoxin 2 (#14,907), anti-TRXR1 (#15,140), anti-Prdx1 (#8499), anti-Prdx2 (#46,855) anti-GAD1 (#41,318), anti-*β*-actin (#3700), anti-PSD95 (#36 233), anti-synaptophysin (#36 406), anti-GAPDH (#5174), anti-lamin b1 (#13,435), anti-MAP2 (#8707) and secondary antibodies, HRP-conjugated anti-rabbit (#7076S), HRP conjugated anti-mouse (7074S), anti-rabbit IgG (H + L), F(ab')2 fragment (Alexa Fluor® 594 Conjugate) (#8889), anti-mouse IgG (H + L), F(ab')2 fragment (Alexa Fluor® 488 Conjugate) (#4408s), ProLong Gold Antifade with DAPI (#8961) and protease/phosphatase inhibitor cocktail (#5872) were purchased from Cell Signaling Technology (Danvers, MA, USA). Anti-NMDAR1 (ab109182), anti-3Ntyr (ab110282), anti-Homer 1 (ab184955), anti-SLC32A1/VGAT (1b235952), and anti-Nrf2 (ab92946) antibodies were purchased from Abcam (Cambridge, UK). Paraformaldehyde solution 4% in PBS (sc-281692), Anti-Trx1 (sc-13526), anti-Trx (sc-271281), and anti-Shank3 (sc-377088) antibodies were acquired from Santa Cruz Biotechnology (Dallas, TX, USA). Thioredoxin Fluorometric Activity Assay Kit (500228) was purchased from Cayman Chemical (Michigan, USA). PX-12 (HY-13734) was purchased from BIOTAG (Kfar Yona, Israel). Other general chemicals were purchased from Sigma-Aldrich (St. Louis, MO, USA) and Bio-Rad Laboratories (Haifa, Israel).

### Animal Experimental Design

*Shank3*^*Δ4−22*^ (Strain #:03 2169) (Drapeau et al. 2018) and C57BL/6J (Strain #:000664) were purchased from the Jackson Laboratory (Farmington, CT, USA). The *Shank3*^*Δ4−22*^ mouse strain is characterized by the deletion of exons 4–22 of the gene encoding the SH3 and multiple ankyrin repeat domains 3 (Shank3). We mated hetero-male *Shank3*^*Δex4−22*^ and hetero-female *Shank3*^*Δex4−22*^ mice. Male *Shank3*^*Δex4−22*^ and their WT littermates were employed in this study for the biochemical assays and behavioral experiments. The animals were kept at room temperature (23 °C) in a 12-h light/dark cycle and fed ad libitum with standard mouse chow and water. All animal experiments were performed according to guidelines set by the Institutional Animal Care and Use Committee (Suckow and Lamberti 2017) and the standards outlined by the Association for Assessment and Accreditation of Laboratory Animal Care International (Gettayacamin and Retnam 2017) (MD-20–16049-3).

### Pharmacological Interventions

The mice were injected intraperitoneally (IP) daily for 10 days. Control mice were injected with the corresponding vehicle (saline). A Trx1 inhibitor, PX-12 (Zheng et al. 2018), was administered to WT and *Shank3*^*Δ4−22*^ mutant mice at the dose of 12 mg/kg (Zheng et al. 2018; Bruycker et al. 2016; Welsh et al. 2003). The following groups of mice were used in this study: (1) WT (*Shank3* WT littermate mice treated with the vehicle), (2) WT + PX-12 (*Shank3* WT littermate mice treated with PX-12), (3) KO (*Shank3*^*Δ4*−22^ KO mice treated with the vehicle), and (4) KO + PX-12 (*Shank3*^*Δ4*−22^ KO mice treated with PX-12).

### Brain Dissection and Isolation

Male mice were used in this study. One hour after the last injection, the animals were euthanized by isoflurane inhalation. The brains were extracted and immediately snap-frozen in liquid nitrogen. The extracted brains were stored at − 80 °C for further analysis. On the experiment day, the entire cortex was isolated (Hamoudi et al. 2022).

### Cell Cultures

SH-SY5Y cells were obtained from the American Type Culture Collection (Manassas, VA, USA) and maintained in a 1:1 mixture of Ham’s F-12 and Dulbecco’s modified Eagle’s medium (DMEM, Thermo Fisher Scientific, Waltham, MA, USA) supplemented with 10% fetal bovine serum, 2 mM l-glutamine, and 1% penicillin‑streptomycin in a humidified atmosphere at 37 °C and 5% CO_2_. Mutant SH-SY5Y cells were generated at our lab using CRISPR-Cas9 technology.

RNAs were designed using the genetic perturbation platform (GPP) sgRNA designer of Broad Institute (Cambridge, MA, USA). sgRNAs were cloned into lentiCRISPR v2 (Addgene #52,961). sgRNAs sequences were used are as follows:

**Table:** 

gRNA	Target gene	Sequence (5′-3′)
humanSHANK3-F1	SHANK3	caccgGAGCACCTCGATGCAAGACC
humanSHANK3-R1	SHANK3	aaacGGTCTTGCATCGAGGTGCTCc
humanSHANK3-F2	SHANK3	caccgTCCACGACCACGCTCAGCTG
humanSHANK3-R2	SHANK3	aaacCAGCTGAGCGTGGTCGTGGAc

For lentiviruses production, a mixture of sgRNA-lentiCRISPR v2, pCMV-dR8.2 dvpr (addgene #8455), and pCMV-VSV-G (addgene #8454) was transfected to HEK293T cells in a respective ratio of 3:2:1. Polyethylenimine** (**PEI) was used as a transfection agent. Following 48 h, lentivirus-containing media was collected, mixed with polybrene, added to the cells, and incubated for 24 h. Transduced cells were selected with 0.5 µg/ml puromycin for 72 h. Then, cells were subcloned in a 96-well plate. Single-cell clones were expanded and screened by immunoblotting of the Shank3 protein.

### Western Blots

Cortex tissues were homogenized on freshly made RIPA buffer (0.15 mM NaCl, 1.3 M glycerol, 16.5 mM Triton X-100, 25 mM NP-40, 35 mM sodium dodecyl sulfate, 0.05M Tris, 1 mM EDTA, 1 mM EGTA, 50 Mm sodium fluoride, 10 mM phenylmethylsulfonyl fluoride (PMSF), 50mM sodium deoxycholate, pH 7.4) supplemented with 1% protease/phosphatase inhibitor cocktail. Homogenization was performed on ice using a Teflon pestle and a Jumbo stirrer (Thermo Fisher Scientific, Waltham, MA, USA). The resultant homogenate was centrifuged at 17,000g for 45 min at 4 °C. The supernatant was stored at − 80 °C for further analysis. Protein quantification of the supernatant was carried out using BCA protein assay. Then, samples were prepared from the tissue lysate using 2X Laemmli buffer with 5% 2-mercaptoethanol and heated at 95 °C for 10 min. The samples were subjected to polyacrylamide gel electrophoresis and subsequently transferred to polyvinylidene fluoride (PVDF) membrane (Bio-Rad Laboratories) using semi-dry transfer. Non-specific sites were blocked by either 5% dried skimmed milk or 5% bovine serum albumin (BSA) in Tris-buffered saline with Tween 20 (TBST), containing 135 mM NaCl, 50 mM Tris, and 0.1% Tween 20, pH 7.4 for 2 h at room temperature. Following incubation, the PVDF membranes containing transferred proteins were washed three times for 10 min each. Next, the membranes were incubated with the primary antibody (1:1000 in 5% BSA) overnight at 4 °C. After this, the membranes were washed three times for 10 min each, followed by incubation with the secondary anti-mouse/rabbit HRP antibody (1:10,000 in TBST). The membranes were washed three more times for 10 min each. Specific binding of the protein of interest was detected using enhanced chemiluminescence (ECL) substrate (Bio-Rad Laboratories). The bands were visualized using the Bio-Rad ChemiDoc imaging system (Hercules, CA, USA). For quantification, we analyzed the gray value of the protein bands by using Bio-Rad Image Lab software. The optical density of the protein bands was normalized for that of the corresponding internal control, β-actin, or GAPDH.

### Primary Cell Culture

Pups were extracted from pregnant WT and KO female mice (embryonic days E17-18); the cortices were isolated, placed in DMEM, and incubated with trypsin and EDTA at 37 °C for 15 min. Then, neurons were pipetted for dissociation and seeded on poly-D-Lysine coated glass in six-well plates. The cells were cultured in a neurobasal medium (Gibco) (penicillin–streptomycin, B27 supplement) in a humidified incubator at 37 °C, 95% air, and 5% CO_2_ for 14 days. To avoid glial cell proliferation, one day after culturing the cells, half of the media volume was replaced with fresh neurobasal media with 2 µM cytosine-*β*-d-arbinofuranoside (Sigma-Aldrich) every 3rd day until day 7.

### Immunocytochemistry

One milliliter of 4% paraformaldehyde solution was applied for 15 min to fix the cells. Following fixation, the cells were gently washed three times with phosphate-buffered saline (PBS) for 10 min each. The cells were then incubated with 0.4 ml of blocking solution (1% BSA, 1% goat serum, and 0.1% Triton X-100) for 2 h at room temperature. After blocking, the cells were again gently washed with PBS three times for 10 min each. Next, the cells were incubated with the primary antibody overnight at 4 °C. After overnight incubation, the cells were washed with PBS three times for 10 min each. The cells were then incubated with secondary antibodies, anti-rabbit Alexa Fluor 594 (1:1000), and anti-mouse Alexa Fluor 488 (1:1000), for 2 h at room temperature. After secondary incubation, the cells were washed three times with PBS for 10 min each. The coverslips were left to dry overnight, and the cells were then mounted with the nuclear fluorescent probe DAPI. Finally, the cells were observed at × 100 magnification using a Nikon confocal microscope.

### Subcellular Fractionation and Sample Preparation for the Nuclear Translocation Assay

The subcellular fractionation was performed according to Abcam instruction (Dr. R. Pattern). Cortex tissues were homogenized on freshly made subcellular fractionation buffer (250 mM Sucrose, 20 mM HEPES, 10 mM KCL, 1.5 mM MgCl2, 1 mM EDTA, 1 mM EGTA, pH 7.4) supplemented with 1% protease/phosphatase inhibitor cocktail. The homogenate was pipetted with the P1000 pipette following the P200 pipette and passed consecutively through the 18-G and then 25-G needle. The resulting homogenate was centrifuged at 720*g* for 5 min at 4 °C. The pellet (nuclear fraction) was solubilized in the subcellular fractionation buffer, passed through a 25-G needle, and centrifuged at 1500*g* for 10 min at 4 °C. Then, the pellet was solubilized with RIPA buffer, sonicated, and stored at − 80 °C for future use. The supernatant was extracted and centrifuged at 10,000*g* for 15 min at 4 °C. The resultant supernatant (cytosolic fractions) was extracted and stored at − 80 °C for further analysis.

The protein quantification of the thawed samples containing cytosolic and nuclear fractions was measured using the BCA protein assay. The protein concentration of each sample (both nuclear and cytosolic) was adjusted to 2 mg/ml using RIPA buffer. Twenty micrograms of protein from each sample was used for western blot analysis. The blot contained proteins from all nuclear and cytosolic samples for direct comparison. GAPDH and Lamin B were used as a loading control. The band intensity ratio of nuclear-to-cytosolic fractions was calculated for each sample.

### Thioredoxin Fluorometric Activity Assay

SH-SY5Y and SH-SY5Y *SHANK3* KO cells were collected (around 5 × 10^6^ cells) by centrifugation at 1,000*g* for 10 min at 4 °C. The cell pellet was then homogenized in 50 µl of cold buffer (100 mM Tris, 1 mM EDTA, pH 7.5), supplemented with a 1% protease/phosphatase inhibitor cocktail. The homogenate was then centrifuged at 10,000*g* for 15 min at 4 °C. The supernatant was extracted and stored at − 80 °C for further use. Prior to the experiment, BCA protein quantification was carried out on the lysates. The protein concentration was adjusted to 0.5 mg/ml. All substrates and samples were prepared according to the Thioredoxin Fluorometric Activity Assay Kit (Cayman Chemical, Michigan, USA) protocol. The relative fluorescence units (RFU), as a Trx activity index, were measured and calculated according to the assay manufacturer protocol (Montano et al. 2014).

### Behavioral Tests

*Shank3*^*Δ4−22*^ KO mice and their WT littermates were treated with PX-12 as described in the “Pharmacological Interventions” section. The corresponding controls were treated with the vehicle. The tests were started on day 5 of the treatments and continued for 5 consecutive days. All behavioral experiments were recorded with a video camera, and data were analyzed by the video tracking software with AI-assisted tracking (Ethovision XT 16, Noldus Information Technology BV) by tracking the mouse movements across the test field (a white square plastic arena, 60X60 cm) in two dimensions. Movements of the three points (tail, center, and nose) were tracked during the recording. Interaction time in all the tests was calculated for the nose tracking point. This protocol was used for all the behavioral tests. The following behavioral tests were performed:

#### The Novel Object Recognition Test

This test was conducted to assess cognition memory and learning (Lueptow 2017). One day before the testing, each mouse was allowed a 5-min habituation period to freely explore the two identical objects placed on each side of the arena. One of the two objects was changed to a novel one on the experiment day. Each mouse was given 5 min to explore the arena. The time spent by the mouse interacting with the novel and the familiar object was reported.

#### The Three-Chamber Sociability Test

To evaluate sociability, the three-chamber test was conducted (Silverman et al. 2010). The arena was divided into three chambers using two opaque partitions. Two empty cages were placed on each of the side chambers. One day before the experiment, a test mouse was placed in the central chamber and given a 10-min habituation period. On the experiment day, one mouse was placed in one of the empty cages. The test mouse was placed in the central chamber and given 10 min to explore the arena. The time spent by the test mouse interacting with the empty cage and the cage with the mouse was reported.

#### The Three-Chamber Social Memory Test

To evaluate preference for social novelty and social memory, the three-chamber social memory test was conducted (Silverman et al. 2010). On the experiment day, the familiar mouse (the mouse used in the three-chamber sociability test) was placed in one of the cages. A novel mouse was placed in the other cage. The test mouse used in the sociability test was introduced again to the central chamber and given 10 min to explore the arena. The time spent interacting with the novel and familiar mouse in the cages was reported.

### Statistical Analysis

Results are expressed as mean ± SEM. Statistical analysis was performed using GraphPad Prism 10.0 software (La Jolla, CA, USA). Data between the two groups was performed using a two-tailed unpaired Student’s *t*-test. Statistical analysis of data from multiple groups was performed using a one-way ANOVA test with Tukey multiple comparison post hoc. A two-way ANOVA test with Tukey post hoc test was used for multiple comparisons in the behavioral tests. *P* < 0.05 was considered statistically significant.

## Results

### Alteration in the Levels of Trx Redox Proteins in the Cortex of the *Shank3* KO Mice

**Fig. 2:**
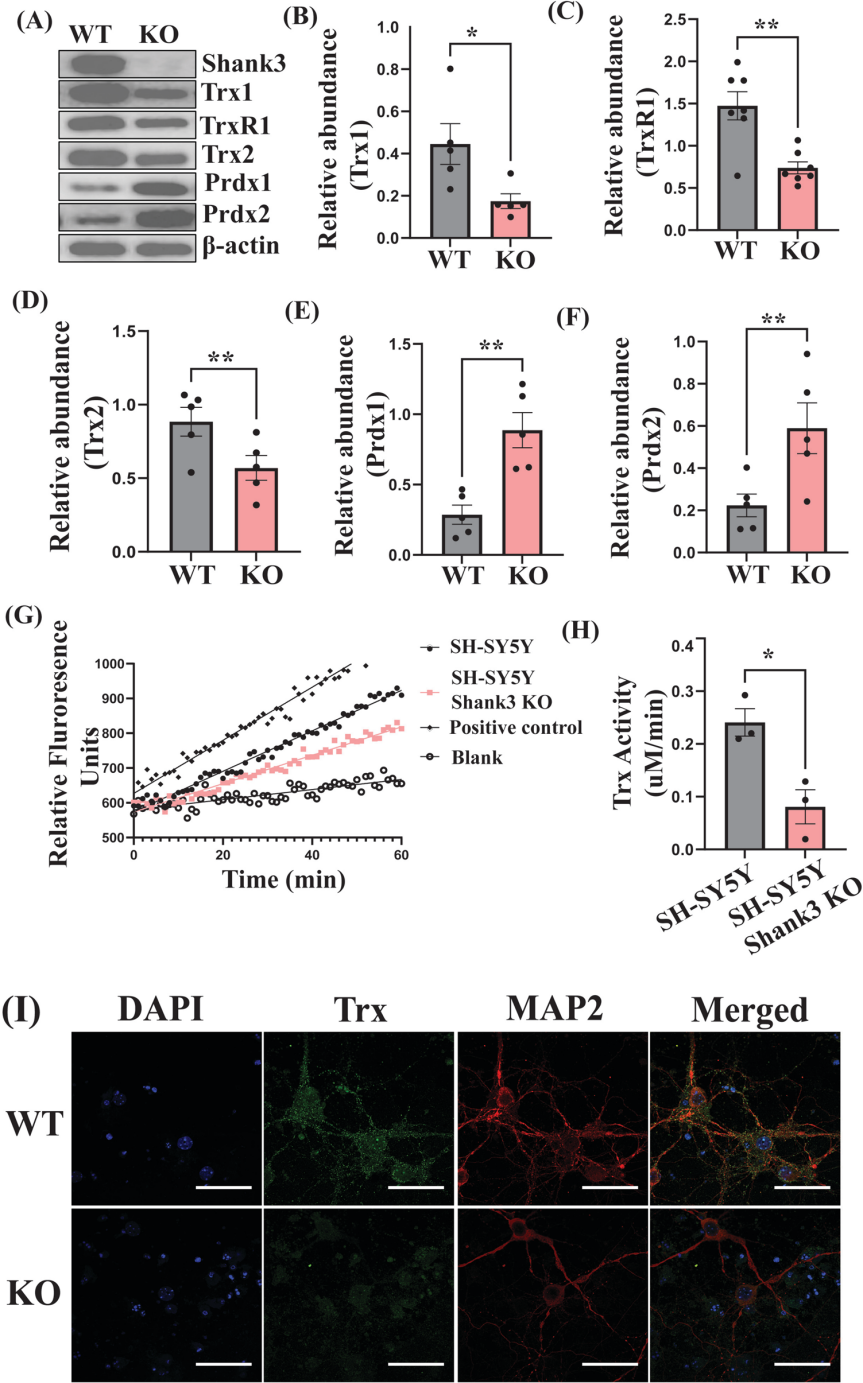
The effect of the *Shank3* mutation on the levels of Trx and Prdx system proteins in the mouse cortex and primary cortical neurons, and Trx activity in the SH-SY5Y *SHANK3 KO* cells. **A** Representative western blots (WB) for Trx1, Trx2, TrxR1, Prdx1, and Prdx2 in the cortex tissues of WT and KO mice. **B–F** Statistical analysis of the relative abundance of Trx1 (*n* = 5), Trx2 (*n* = 5), TrxR1 (*n* = 7), Prdx1 (*n* = 5), and Prdx2 (*n* = 5), respectively, in the cortex of WT and KO mice. **G** A scatter plot showing average RFU for Trx activity in SH-SY5Y and SH-SY5Y *SHANK3* KO cells.** H** Statistical analysis of the Trx activity in SH-SY5Y and SH-SY5Y *SHANK3* KO cells (*n* = 3). **I** Representative confocal images of the fluorescence of Trx, MAP2 (a marker of neuronal differentiation), and DAPI (a marker of nuclei) in the primary cortical neuronal cell cultures derived from WT and *Shank3 *KO mice. The image was captured at × 100 magnification. The scale bar = 50 µm. *P < 0.05, **P < 0.01

To determine whether *Shank3* mutation leads to alteration in the expression of Trx redox proteins, we compared the levels of Trx1, TrxR1, Trx2 (the mitochondrial isoform of thioredoxin (Watson et al. 2004)), Prdx1, and Prdx2 in the cortices of 6-week male *Shank3* wild-type (WT) and *Shank3*Δ^*4−22*^ KO (KO) mice. We found a significant decrease in the protein levels of Trx1, TrxR1, and Trx2 (Fig. 2B–D) in the KO mice. A significant increase in the levels of Prdx1 and Prdx2 was also observed in these mice compared to their WT counterparts (Fig. 2E and F). Then, the Trx activity was evaluated in SH-SY5Y and SH-SY5Y *SHANK3* KO cells. As shown in Fig. 2G, we observed a reduction in the RFU of the mutant cells compared to the normal SH-SY5Y cells, indicating the reduced Trx activity (µM/min) in the mutant cells (Fig. 2H). To validate our data at the organism level, we compared the confocal images of primary cortical neurons from WT and KO mice. The reduction in Trx levels in the cortex of KO mice was confirmed by the weakened fluorescence of Trx in the primary cortical neuron cell culture of these mice compared to WT (Fig. 2I).

### Alteration in the Nuclear Translocation of Trx1 and Prdx1 in the *Shank3* KO Mice

**Fig. 3:**
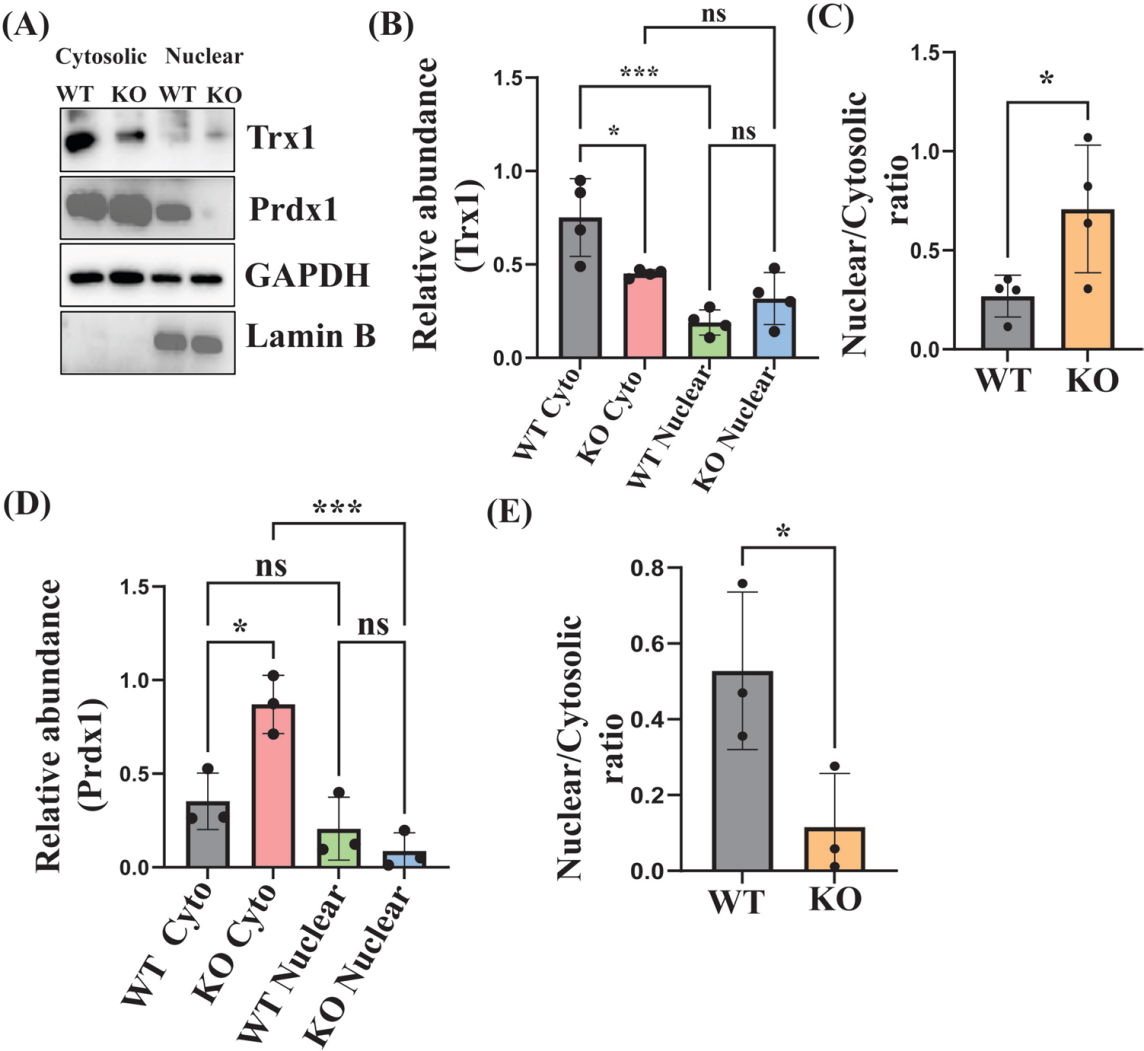
The cytosolic and nuclear levels of Prdx1 and Trx1, and their translocation to the nucleus in the cortex of *Shank3* KO and WT mice. **A** Representative WB for Trx1 in the cytosolic (Cyto) and nuclear (Nuc) fractions of the cortex of WT and KO mice. GAPDH and Lamin B were used as a reference for the cytosolic and nuclear protein loading, respectively.** B** Statistical analysis of the relative abundance of Trx1 in the cytosolic and nuclear fractions in the cortex of WT and KO mice (*n* = 4). **C** Statistical analysis of the nuclear/cytosolic fractions ratio of the Trx1 relative abundance in the cortex of WT and KO mice (*n* = 4).** D** Statistical analysis of the relative abundance of Prdx1 in the cytosolic and nuclear fractions of the cortex of WT and KO mice (*n* = 3).** E** Statistical analysis of the nuclear/cytosolic ratio of the Prdx1 relative abundance in the cortex of WT and KO mice (*n* = 3). GAPDH and Lamin B were used as a reference for the cytosolic and nuclear protein loading, respectively. *P < 0.05, **P < 0.01, ***P < 0.001; ns, non-significant

To determine whether Trx1/Prdx1 translocation occurred in the *Shank3* mouse model of ASD, we measured cytosolic and nuclear levels of these enzymes in WT and KO mice. Significantly higher levels of Trx1 were observed in the cytosol compared to the nucleus in WT mice. In contrast, this difference was not observed in the KO mice (Fig. 3B). A significant reduction in the cytosolic levels of Trx1 was found in KO compared to WT mice (Fig. 3B). KO mice had a significantly higher nuclear/cytosolic fractions ratio of Trx1 compared to WT mice (Fig. 3C).

In WT mice, the levels of Prdx1 in the cytosol were insignificantly different compared to the nucleus (Fig. 3D). Meanwhile, we observed significantly higher levels of Prdx1 in the cytosol fraction compared to the nucleus in the cortex of the KO mice (Fig. 3D). In addition, the cytosolic levels of Prdx1 were about twofold higher in KO mice compared to WT mice, and the nuclear/cytosolic ratio of Prdx1 was considerably lower in KO compared to WT mice (Fig. 3E).

### Thioredoxin 1 Inhibition Led to Elevated Nitrosative Stress and Alterations in Synaptic Proteins Similar to *Shank3* Mutant Mice

**Fig. 4:**
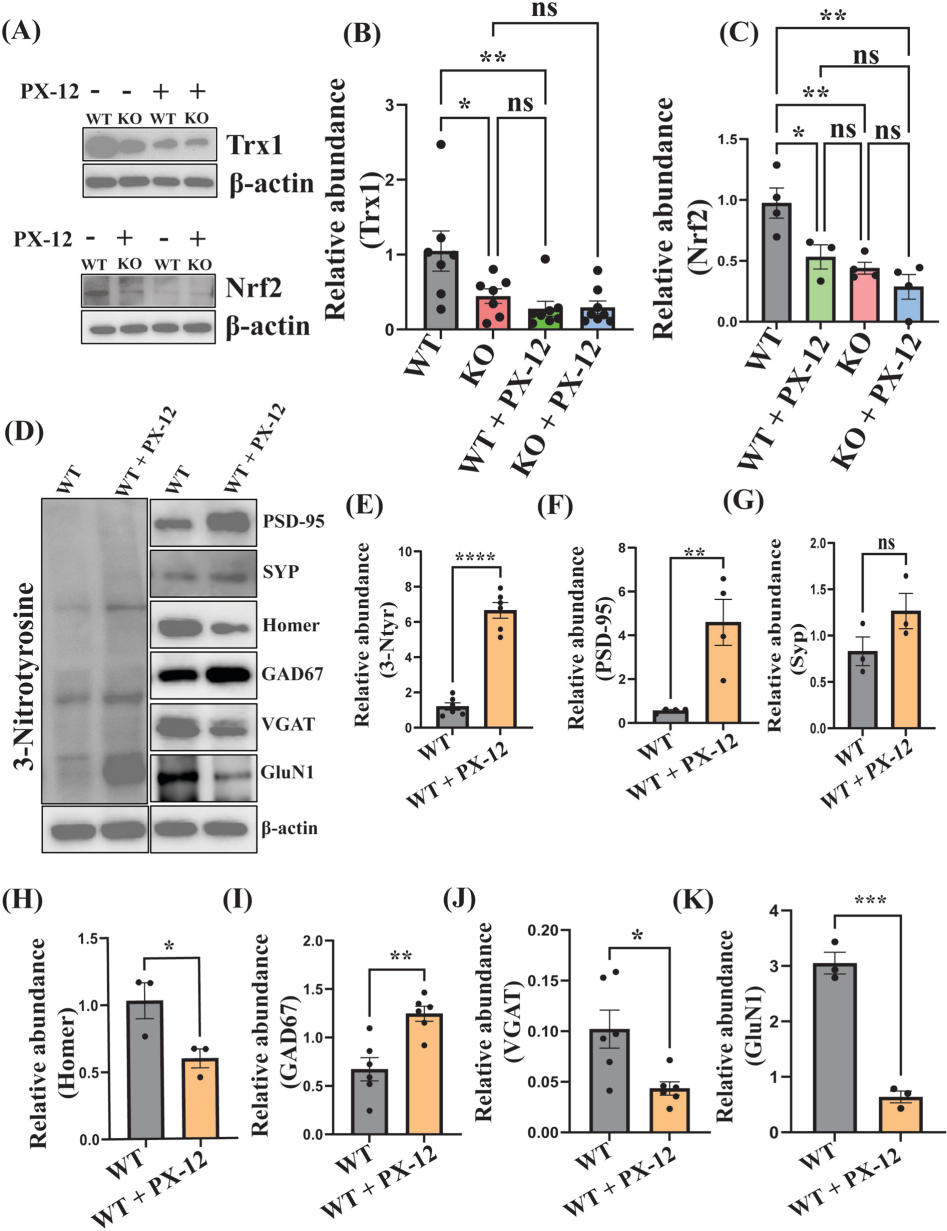
Effects of Trx1 inhibition with PX-12 on the levels of Trx1, Nrf2, 3-Ntyr, and the molecular markers of synaptic phenotype in the cortex of WT and *Shank3* KO mice. **A** Representative WB for Trx1 and Nrf2 from the cortex of WT, KO, WT + PX-12, and KO + PX-12 mice. β-actin was used as a reference for protein loading. **B** Statistical analysis of the relative abundance of Trx1 in the cortex of WT (*n* = 6), KO (*n* = 6), WT + PX-12 (*n* = 7), and KO + PX-12 (*n* = 7) mice.** C** Statistical analysis of the relative abundance of Nrf2 in the cortex of WT (*n* = 4), KO (*n* = 3), WT + PX-12 (*n* = 4), and KO + PX-12 (*n* = 4) mice. **D** Representative WB for the molecular markers of nitrosative stress (3-nitrotyrosine (3-Ntyr)) (*n* = 6), synaptic phenotype (PSD95) (*n* = 4), synaptophysin (SYP) (*n* = 3), Homer (*n* = 3)), GABAergic system (GAD67) (*n* = 6) and (VGAT) (*n* = 6)), and glutamatergic system (GluN1) (*n* = 3) in the cortex of WT and WT + PX-12 mice. β-actin was used as a reference for protein loading. **E, F** Statistical analysis of the relative abundance of 3-Ntyr, PSD95, SYP, Homer, GAD67, VGAT, and GluN1, respectively, in the cortex of WT, and WT + PX-12 mice. *P < 0.05, **P < 0.01, ***P < 0.001, ****P < 0.0001; ns, non-significant

To study the involvement of Trx1 on the autistic phenotype, we inhibited its activity using PX-12 (12 mg/kg). A significant reduction in the levels of Trx1 was found in the cortex of WT mice treated with PX-12 (WT + PX-12 group) compared to the non-treated ones. However, this difference was not observed between the treated and non-treated KO mice (Fig. 4B).

Nrf2 is the master regulator of the endogenous antioxidant proteins (Vomund et al. 2017). The levels of Nrf2 were significantly decreased in the cortex of PX-12-treated WT mice compared to the non-treated ones. In contrast, the treatment of the KO mice with PX-12 (KO + PX-12 group) had no significant effect on Nrf2 levels. Meanwhile, the baseline levels of this protein were twice as low in the KO mice as in their WT littermates (Fig. 4C). Interestingly, Trx1 inhibition with PX-12 in WT mice led to a significant increase in the levels of Prdx1 and Prdx2 in the mouse cortex (Suppl. Figure 1B and C), similar to the effect of the *Shank3* mutation on the levels of this enzyme (Fig. 2E and F).

We suggested that PX-12 administration might inhibit Trx1 denitrosylating function, leading to elevated nitrosative stress, which could affect synaptic phenotype. To check this hypothesis, we evaluated the effect of PX-12 on the levels of 3-Ntyr (a marker of nitrosative stress (Bandookwala and Sengupta 2020)) and synaptic proteins, including the postsynaptic density protein 95 (PSD95), the synaptophysin (SYP), the PSD scaffold protein Homer, the GABAergic markers glutamate decarboxylase 67 (GAD67, also referred as GAD1), the vesicular GABA transporter (VGAT), and the glutamatergic marker NMDA receptor subunit (GluN1) in WT mice. A sixfold increase in the levels of 3-Ntyr was observed in the cortex of the PX-12-treated vs. non-treated WT mice (Fig. 4E). The levels of PSD95 were also noticeably increased, while the SYP levels were insignificantly changed in the treated mice (Fig. 4F and G, respectively). The levels of Homer were significantly reduced (Fig. 4H), and the levels of GAD67 were considerably elevated, while the levels of the VGAT were halved in WT + PX-12 compared to WT mice (Fig. 4H–J, respectively). GluN1 levels were also visibly decreased in the cortices of treated mice (Fig. 4K). Key changes found in WT + PX-12 mice are similar to the changes observed in *Shank3* mutant mice (Tripathi et al. 2023).

### Thioredoxin 1 Inhibition Induced an ASD-Like Behavioral Phenotype

**Fig. 5:**
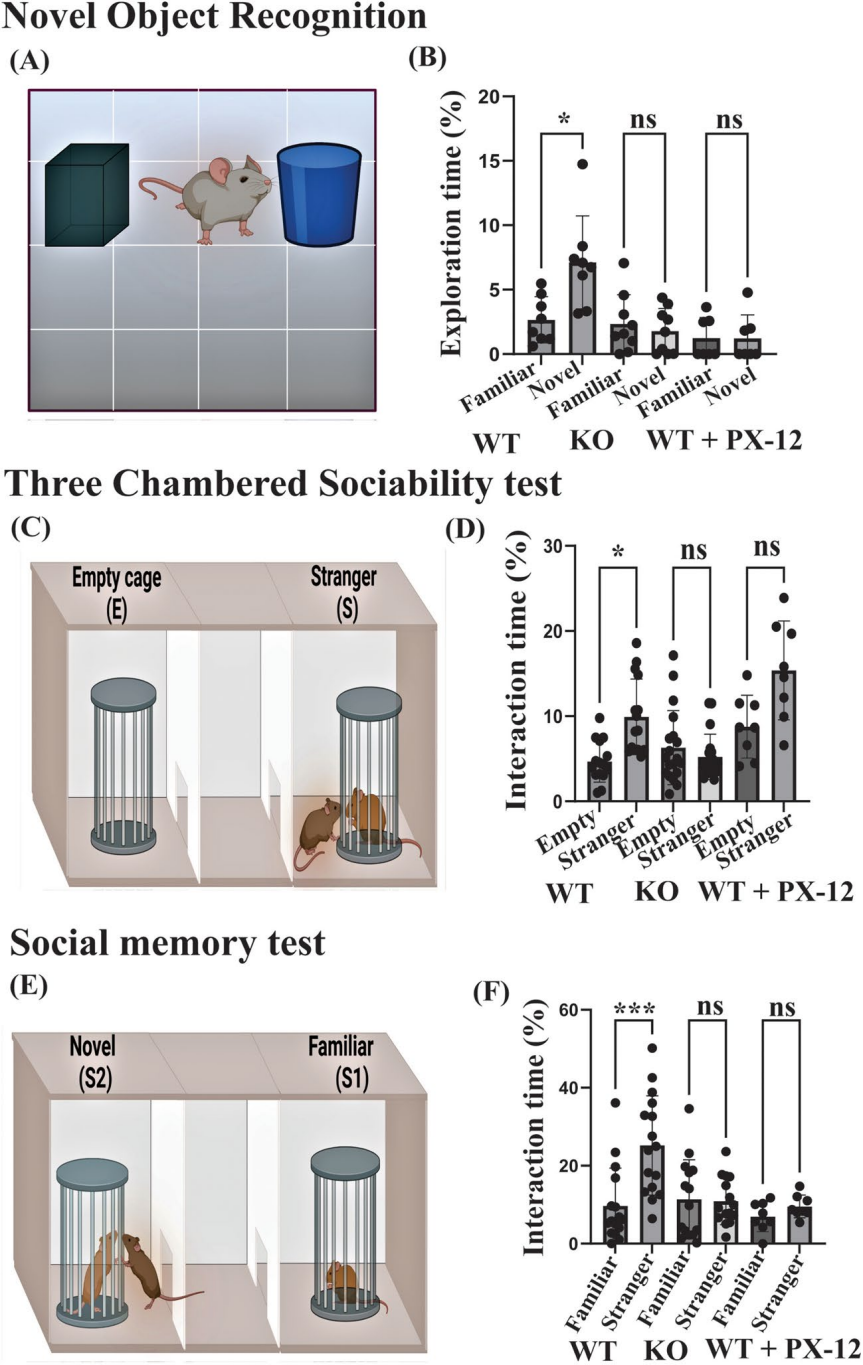
PX-12 treatment induces autistic behavior abnormalities in WT mice similar to those in *Shank3 KO* mice. **A**, **C**, **E** Schematic representation of the novel object recognition test, three-chamber sociability test, and three-chamber social memory test, respectively. **B** Statistical analysis of the time spent by the test mouse for interaction with novel and familiar objects in WT (*n* = 8), KO (*n* = 9), WT + PX-12 (*n* = 7), and KO + PX-12 (*n* = 8) groups of mice. **D** Statistical analysis of close interaction time spent by the test mouse with a caged stranger mouse and an empty cage in WT (*n* = 15), KO (*n* = 15), WT + PX-12 (*n* = 8), and KO + PX-12 (*n* = 8) groups of mice. **F** Statistical analysis of the time spent by the test mouse for interaction with a caged stranger and familiar mouse in WT (*n* = 19), KO (*n* = 19), WT + PX-12 (*n* = 8), and KO + PX-12 (*n* = 8) groups compared to familiar mice. *P < 0.05, ***P < 0.001; ns, non-significant

To link our biochemical and cellular findings to behavioral manifestations of ASD, we tested the effects of Trx1 inhibition on the behavior of WT and KO mice. First, we performed the novel object recognition test (Fig. 5A). The test showed that WT mice exhibited a notably higher interest in the novel object than the treated mice, spending a significantly longer time interacting with the novel rather than the familiar object. In contrast, there was no difference between the time spent with the novel and familiar objects in KO and WT + PX-12 mice (Fig. 5B). Mice of the KO + PX-12 group did not show a significant preference towards novel or familiar objects (Suppl. Figure 1D). Then, we performed the three-chamber sociability tests (Fig. 4C) to assess cognition in the form of sociability and social memory. Mice normally prefer to spend a longer time with a stranger mouse. This was observed in the WT mice who favored interacting with the mouse placed in the cage more than the empty cage. They spent a significantly longer time interacting with the caged mouse. Meanwhile, in KO and WT + PX-12 mice, there was no significant difference between the interaction time with the mouse in the cage and the empty cage (Fig. 5D). Mice of the KO + PX-12 group did not exhibit any significant preference between the mouse cage and the empty cage (Suppl. Figure 1E). Lastly, we performed the three-chamber social memory test (Fig. 5E). Normal mice usually prefer to interact longer with the intruder mouse than with the familiar mouse (Silverman et al. 2010). The test results showed that this was a characteristic of WT mice. These animals spent significantly longer time interacting with the novel mouse placed in the cage. Contrary to WT mice, KO and WT + PX-12 did not show any clear preference for interacting with the novel and the familiar mouse (Fig. 5F). No significant difference was found between KO and KO + PX-12 groups in this test (Suppl. Figure 1F).

## Discussion

The human brain’s weight accounts for 2% of the body’s weight but consumes 20% of the whole body’s oxygen. This high oxygen demand, high levels of transition metals, and unsaturated fatty acids, coupled with low levels of some important antioxidant enzymes, such as superoxide dismutase, catalase, and glutathione peroxidase (Dringen et al. 2000), make the brain very vulnerable to oxidative/nitrosative stress when ROS and RNS production exceeds the ability of the endogenous antioxidant systems to neutralize these species (Silva-Adaya et al. 2014; Raymond et al. 2014). Oxidative stress is an important pathogenic factor of both neurodegenerative (Chen et al. 2012; Barnham et al. 2004; Singh et al. 2019) and neurodevelopmental disorders including ASD (Bjørklund et al. 2020a, 2020b; Nishimura et al. 2021; Membrino et al. 2023).

A major antioxidant system in the brain is the Trx system. Its activity is essential for supporting the redox balance in the nervous system (Silva-Adaya et al. 2014; Raymond et al. 2014; Bjørklund et al. 2022). The Trx system shows cytoprotective functions against oxidative stress by scavenging ROS in cooperation with Trx-dependent peroxidase (Karlenius and Tonissen 2010). Trx and NO are closely interconnected and can influence each other in various ways (Benhar 2015; Benhar et al. 2008; Nikitovic and Holmgren 1996; Qu et al. 2012; Wu et al. 2011; Sengupta and Holmgren 2013; Haendeler 2006). For instance, Trx system plays a major role in denitrosylation of S-nitrosylated proteins (Sengupta and Holmgren 2012). The protective role of this selenoenzyme also includes anti-apoptotic, growth-promoting, and inflammation-modulating effects (Bjørklund et al. 2022). Therefore, the impairments in the Trx system may contribute significantly to neurodevelopmental disorders (Abd-Allah et al. 2020). A few previous studies have reported increased levels of Trx proteins in the blood of autistic children in response to oxidative stress (Abd-Allah et al. 2020; Zhang et al. 2015). In contrast, our results show a significantly reduced Trx activity in SH-SY5Y cells with *SHANK3* gene deletion and depleted Trx1, TrxR1, and Trx2 proteins in the cortex of the *Shank3* mouse model of ASD (Fig. 2). To the best of our knowledge, this is the first report on the downregulation of the Trx system in autistic brain. A possible explanation for the reduced Trx activity in *Shank3* mice could be elevated S-nitrosylation caused by high nitric oxide (NO) levels. This can inactivate Trx (Sengupta and Holmgren 2012), preventing it from activating Nrf2 and consequently leading to reduced transcription of antioxidant ezymes.

It has been found that Trx activity can be regulated by changes in the levels of growth factors, such as the nerve growth factor (Silva-Adaya et al. 2014) and cAMP response element-binding protein (Oberacker et al. 2023). PTMs, including phosphorylation, SNO, and glutathionylation, can affect the activity of the Trx system (Haendeler 2006). The activity of this system can also be modulated by inhibitors that bind to one or more proteins of this system. For example, the thioredoxin-interacting protein can bind to and inhibit the activity of Trx (Nishiyama et al. 1999). Our results show that the levels of Nrf2 in the cortex of *Shank3* KO mice were halved (Fig. 4C). As we mentioned above, it can be speculated that the reduction in the levels of Trx proteins in the *Shank3* KO mice and cells was at least partially caused by the downregulation of Nrf2. Indeed, it has been recently found that the Shank3 protein can induce downstream phosphorylation of Nrf2 at Ser40, leading to Nrf2 translocation to the nucleus and an increase in the antioxidant gene expression (Zhang et al. 2024). Therefore, on the one hand, it can be suggested that the lack of the Shank3 protein in our KO mice decreased the levels of Nrf2 in the nucleus leading to the downregulation of the Trx system proteins despite the increased oxidative stress characteristic of the autistic brain (Bjørklund et al. 2020a, 2020b; Nishimura et al. 2021; Membrino et al. 2023). On the other hand, Sebula et al. (Cebula et al. 2015) have found that TrxR1 can regulate the expression of Nrf2. Other studies have shown that Trx1’s nuclear translocation activates Nrf2, which in turn increases the expression of the antioxidant proteins including Trx1 itself (Hasan et al. 2022). This can explain the decrease in the levels of Trx1 following its inhibition by PX-12. Interestingly, the levels of Prdx1/2 were more than doubled in the cortex lysates of *Shank3* KO mice. Prdxs are ROS-regulating intracellular enzymes (Rhee et al. 2005; Jeong et al. 2020) that can reduce ROS-induced stress and modulate cell redox status under physiological conditions or oxidative stress (Woo et al. 2010; Kim et al. 2022). Hence, the increased expression of Prdx1/2 in the cortex of *Shank3* KO mice could be a compensatory reaction to the increased oxidative stress on the background of the reduced levels and activity of the Trx system. Despite the increased total levels of the Prdx proteins, their levels in the nuclear fraction of the cortices of these mice were significantly reduced (Fig. 3D). Normally, there is a balance between the expression and activity of Trx and Prdx, which protects each other’s redox status (Hansen et al. 2007). However, our results indicate that this feature is lost in the cortex of *Shank3* KO mice, which could increase ROS production in ASD.

Trx1 is mainly located in the cytoplasm, while Trx2 is a mitochondrial protein (Ren et al. 2017). Oxidative stress promotes Trx1 translocation to the nucleus where it modulates the activity of many transcription factors (Zhao et al. 2015). In our experiments, *Shank3* genetic deletion in mice resulted in enhanced Trx1 nuclear translocation (Fig. 3B and C). The exact mechanism of this phenomenon remains obscure. However, a few studies point to the stimulation of the Trx1 nuclear translocation by the inflammation-related proteins. Thus, Wiesel et al. (Wiesel et al. 2000) have found that pro-inflammatory mediators, lipopolysaccharides, and interleukin (IL)-1β promote Trx1 nuclear translocation in rat aortic smooth muscle cells and macrophages. In another recent work on the human colorectal cancer cell lines, Wu et al. (Wu et al. 2023) have shown that IL-6 mediates the translocation of Trx1 to the nucleus. Inflammation is typical of ASD (Siniscalco et al. 2018). Therefore, the Trx1 nuclear translocation in the *Shank3* mouse model of ASD might be stimulated by inflammation. Interestingly, the nuclear Trx1 itself can induce an inflammatory response (Go et al. 2014, 2013), and this could form a positive feedback between the inflammation and the nuclear Trx1 in the autistic brain.

Another possible stimulator of the nuclear translocation of this redox-regulating enzyme might be nitrosative stress. Our previous studies have demonstrated increased NO• levels and a reprogramming of the SNO-proteome in the *Shank3* mouse models of ASD indicating that NO• and protein SNO may play a role in ASD pathology (Amal et al. 2020a, 2020b; Tripathi et al. 2020). Our further works confirmed the increased levels of NO• in *Shank3* KO mice and revealed that this leads to nitrosative stress manifested in the elevation of 3-Ntyr concentration in the brains, SNO of various proteins, particularly of the glutamatergic system, and altered levels of synaptic proteins (Tripathi et al. 2023; Hamoudi et al. 2022). Meanwhile, it has been revealed that the nitrosylation of p21Ras results in Trx-1 accumulation in the nucleus via the activation of ERK1/2 MAP kinases (Hasan et al. 2022).

Inhibition of the Trx system by PX-12 in WT mice caused remarkable similarities with the *Shank3* ASD mouse model used in our experiments. Thus, this treatment resulted in a strong reduction in Trx1, TrxR1, and Trx2 levels, over fivefold increase in 3-Ntyr content, and a significant drop in GluN1 and Homer levels in the cortex (Fig. 4). This means that the pharmacological inhibition of the Trx system in mice led to a dramatically increased nitrosative stress and impairments in the glutamatergic system consistent with the *Shank3* mouse model of ASD (Amal et al. 2020a, 2020b; Tripathi et al. 2023; Hamoudi et al. 2022). Also, Trx1 inhibition caused synaptic deficits in WT mice, similar to the deficits observed in *Shank3* KO mice (Tripathi et al. 2023, 2024; Abdel-Haq et al. 2023). A potential reason for this phenomenon could be the increased oxidative and nitrosative stress due to the loss of Trx1’s reducing and de-nitrosylating functions. Oxidative stress can cause synaptic damage and neuronal death (Vajda 2002; McDougle and Carlezon 2013), and elevated S-nitrosylation of synaptic proteins can alter them (Kartawy et al. 2021, 2020; Hamoudi et al. 2021), contributing to synaptic deficits. Furthermore, these similarities were manifested in ASD-like behavioral deficits which represent the most important features of this disorder. Both *Shank3* KO and PX-12-treated WT mice displayed reduced cognition memory and restricted interest in the novel object recognition test (Fig. 5A and B), lack of sociability, reduced preference for social novelty, and restricted social memory in the three-chamber sociability test (Fig. 5C–F). All these deficits are the core symptoms of ASD (Tong et al. 2024). These results imply that the impairments in the Trx system, in particular, the downregulation of Trx1 and Trx2 in the cortex and disbalance between the Trx and Prdx systems, are implicated in ASD pathology, and the components of these regulators of redox and nitrosylation state might be a target for a future ASD treatment. Interestingly, the treatment of KO mice with PX-12 did not lead to a significant change in the behavioral tests (Suppl. Figure 1 D-F). Neither did this treatment affect the Trx1 or Nrf2 levels in the cortex of KO mice (Fig. 4B and C). The lack of the effects of the pharmacological Trx inhibition in KO mice could be explained by the significantly reduced levels of the Trx proteins already caused by the genetic deletion of the *Shank3* gene, as observed in this study.

It should be noted, however, that Trx activity inhibition in mice does not entirely recapitulate the *Shank3* KO phenotype. Thus, the PX-12 treatment of WT mice resulted in increased levels of PSD95, and GAD67, and a downregulation of VGAT, indicating the increased maturation of glutamatergic (Coley and Gao 2018) and imbalance in the GABAergic (Ma et al. 2016) systems. In contrast, the levels of these proteins in *Shank3* KO mice were significantly decreased (Tripathi et al. 2023).

## Conclusions

This study revealed for the first time a significant downregulation of the components of the Trx system in the cortex of the *Shank3* model of ASD. We also observed a considerable reduction of Nrf2 levels, increased nuclear translocation of Trx1, and upregulation of the Prdx1 in the brains of these mice. The results of this work confirm the involvement of the Trx/Prdx system in the pathology of ASD manifested in oxidative/nitrosative stress, synaptic dysfunction, and behavioral deficits, as shown in the Graphical Abstract. Therefore, components of the Trx system and related proteins may represent novel targets for ASD treatment. These findings might open new horizons in the search for effective ways to identify drug targets. Further research is necessary to unravel the mechanisms underlying these effects.

## Disclaimer

HA is a CSO of Point6 Bio and Neuro-NOS. No funds from both companies were received for this study. All other authors do not hold any competing interests.

## Supplementary Information

Below is the link to the electronic supplementary material.

Supplementary file1 (DOCX 259 KB)

## Data Availability

No datasets were generated or analysed during the current study.
