# Supplementary material for: The Role of Thioredoxin System in Shank3 Mouse Model of Autism
Source: J Mol Neurosci. 2024 Sep 30;74(4):90. doi: 10.1007/s12031-024-02270-y (PMC11457715; doi:10.1007/s12031-024-02270-y)
Supplement: Supplementary file 1 — Supplementary file1 (DOCX 259 KB) [file 12031_2024_2270_MOESM1_ESM.docx]

**Supplementary Information**

**The Role of Thioredoxin System in *Shank3* Mouse Model of Autism**

Wisam Bazbaz, Maryam Kartawy, Manish Kumar Tripathi, Wajeha Hamoudi, Shashank Kumar Ojha, Igor Khaliulin, Haitham Amal*

Institute of Drug Research, School of Pharmacy, Faculty of Medicine, The Hebrew University of Jerusalem.

*Correspondence: Prof. Haitham Amal, [Haitham.amal@mail.huji.ac.il](mailto:Haitham.amal@mail.huji.ac.il)

Supplementary Results

**
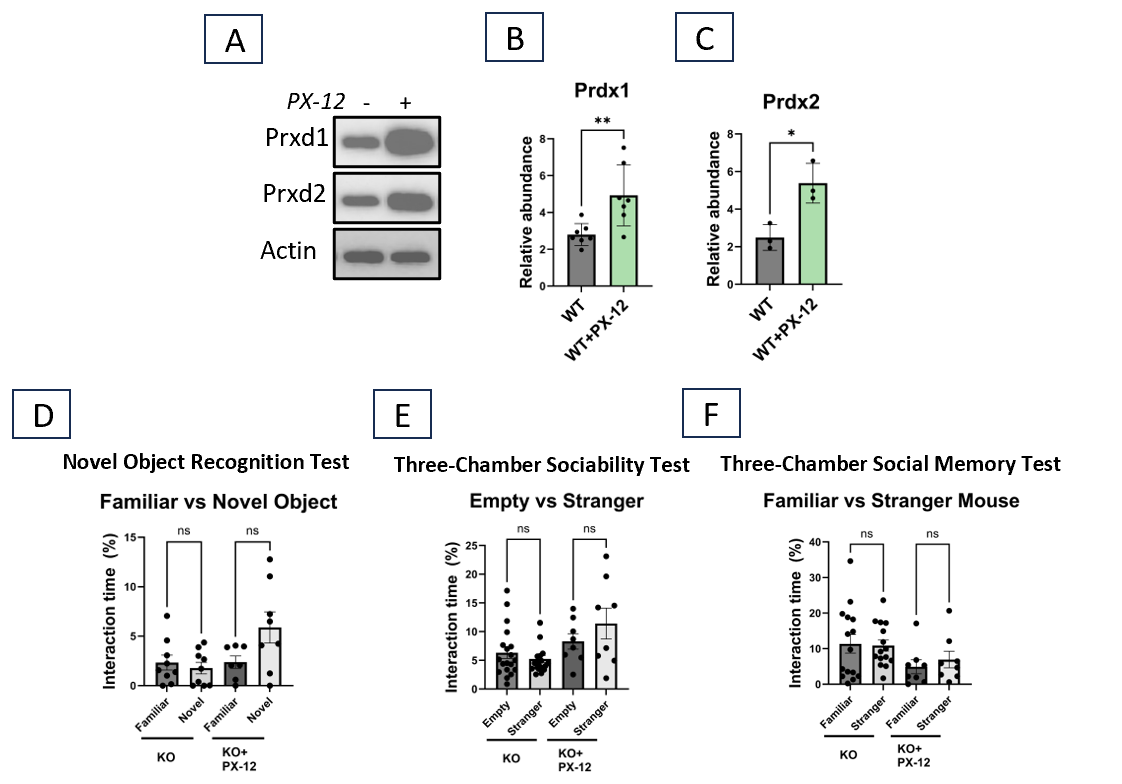
**

**Supplementary Figure 1.** Elevation in the levels of Peroxiredoxin 1 (Prdx1) and Peroxiredoxin 2 (Prdx2) in the cortex tissues of *Shank3* KO (KO) mice compared to wild-type (WT) mice. **A.** Representative WB for Prdx1 and Prdx2 in the cortex of WT treated (+) and non-treated (-) with PX-12. **B** and **C.** Statistical analysis of the relative abundance of Prdx1 (n=7) and Prdx2 (n=7), respectively, in the cortex of WT and WT+PX-12 groups of mice. **D.** Statistical analysis of the time spent by the test mouse for interaction with novel and familiar objects in KO (n=9) and KO+PX-12 (n=8) groups. **E.** Statistical analysis of the time spent by the test mouse for interaction with a caged stranger mouse and empty cage in KO (n=15) and KO+PX-12 (n=8) groups. **F)** Statistical analysis of close interaction time with a caged stranger and familiar mouse in KO (n=19) and KO+PX-12 (n=8) groups. * P < 0.05, ** P<0.01, ns – non-significant.
